# Prospective evaluation of accuracy of HIV viral load monitoring using the Aptima HIV Quant Dx assay with fingerstick and venous dried blood spots prepared under field conditions in Kenya

**DOI:** 10.1371/journal.pone.0249376

**Published:** 2021-04-02

**Authors:** Matilu Mwau, Jeff Danda, Joseph Mbugua, Allan Handa, Jacqueline Fortunko, Andrew Worlock, Sangeetha Vijaysri Nair

**Affiliations:** 1 Kenya Medical Research Institute, Nairobi, Kenya; 2 Hologic, Inc., San Diego, CA, United States of America; Stanford University School of Medicine, UNITED STATES

## Abstract

Quantification of HIV-1 RNA is essential for clinical management of HIV patients. The limited throughput and significant hands-on time required by most HIV Viral load (VL) tests makes it challenging for laboratories with high test volume, to turn around patient results quickly. The Hologic Aptima HIV-1 Quant Dx Assay (Aptima), has the potential to alleviate this burden as it is high throughput and fully automated. This assay is validated for both plasma and dried blood spots (DBS), which are commonly used in resource limited settings. The objective of this study was to compare the performance of Aptima to Abbott RealTime HIV-1 Assay (Abbott RT), which was used as reference. This was a cross-sectional prospective study where HIV VL in finger stick (FS) DBS, venous blood (VB) DBS and plasma, collected from 258 consenting adults visiting 5 medical facilities in Kenya, Africa were tested in Aptima. The results were compared to plasma VL in Abbott RT at the medical decision point (MDP) of 1000 copies/mL and across Aptima assay range. The total agreement at MDP between plasma HIV VL in Abbott RT and plasma, FS and VB DBS tested in Aptima were 97.7%, 92.2% and 95.3% respectively with kappa statistic of 0.95, 0.84 and 0.90. The positive and negative agreement for all 3 sample types were >92%. Regression analysis between VL in Abbott RT plasma and various sample types tested in Aptima had a Pearson’s correlation coefficient ≥0.91 with systematic bias of < 0.20 log copies/mL on Bland-Altman analysis. The high level of agreement in Aptima HIV VL results for all 3 sample types with Abbott RT plasma VL along with the high throughput, complete automation, and ease of use of the Panther platform makes Aptima a good option for HIV VL monitoring for busy laboratories with high volume of testing.

## Introduction

Human immunodeficiency virus (HIV) infection is a major cause of morbidity and mortality globally with more than 60% of HIV-1 infected individuals residing in Africa [1, 2]. As of 2019, 65% of the HIV patients across the world were receiving antiretroviral therapy (ART), which is crucial to protect the patient’s health and slow the progression of the HIV epidemic. Viral load quantitation is a more effective means of monitoring response to ART than clinical assessment and CD4 counts, as it enables accurate detection of treatment failure [3, 4]. The World Health Organization (WHO) strongly recommends the routine usage of viral load (VL) testing for monitoring response to ART including in resource-limited settings [5]. A threshold of 1000 c/mL of HIV-1 VL is recommended by the WHO for monitoring effectiveness of ART as the risk of HIV transmission is low with VL below this threshold [5].

In 2018, 1.6 million Kenyans were living with HIV with over 1 million people receiving ART [2]. Like in most resource limited countries, effectiveness of ART is monitored in Kenya using VL testing of not only plasma but also DBS specimens. DBS is a good option especially in rural and resource limited settings as it can be prepared by technicians with limited training and fewer equipment’s, because phlebotomy and centrifugation of blood are not required for preparation of FS DBS. DBS is non biohazardous when dry and reduces the cost of VL monitoring because it can be stored and shipped at room temperature. The ease of DBS collection and transport facilitates decentralization of specimen collection, increasing access to VL monitoring and enabling more timely monitoring for treatment failure, which are crucial for reducing HIV transmission and emergence of drug resistance.

All the HIV VL testing in Kenya is conducted in 9 centralized laboratories [6] which test large numbers of DBS and plasma specimens mostly on Abbott m2000 and the Roche Cobas Ampliprep/ Cobas Taqman (CAP/CTM) platforms. Since these medium throughput platforms [7], are used to run a large number of tests, backlogs are quite common. As countries across Africa scale up VL testing to achieve UNAIDS 90-90-90 targets more high throughput options are required to decrease sample backlogs and improve turn-around times for results.

The Aptima HIV-1 Quant Dx Assay (Aptima Assay) is an in vitro nucleic acid amplification test, that is run on the high throughput fully automated Panther platform. It is intended for the detection and quantification of Human Immunodeficiency Virus type 1 (HIV-1) in human plasma, and DBS specimens [8, 9]. Several publications demonstrate equivalent performance of the Aptima Assay with other commercially available assays, but most of these were conducted by testing plasma specimens from HIV patients in Europe and the United States [10–20]. Unlike the United States and Europe where the predominant HIV subtype is subtype B, Africa has the greatest genetic diversity of HIV-1 with Kenya having high level of infection with recombinants, subtype A, C and D [21, 22].

The limited published information on the performance of DBS specimens in the Aptima Assay were generated by testing DBS prepared with venous blood (VB DBS) collected from patients in the United States and Australia [23–25]. Of these only one small study with less than 100 patients compares the VL of VB DBS and plasma at the MDP of 1000 copies/mL [25]. Although Finger Stick (FS) DBS is widely used for VL monitoring in Africa there is currently no published information available on the performance of FS DBS tested in Aptima for VL monitoring of patients on ART.

The purpose of this study was to evaluate the performance of FS DBS along with VB DBS and plasma tested in the Aptima Assay for monitoring response to ART, by comparing VL in these sample types to the Abbott RT plasma result, used as reference. Since the testing was performed with clinical specimens collected from multiple sites in Kenya, this also serves to assess whether the Aptima quantification of the diverse HIV subtypes seen in Kenya is equivalent to that of Abbott RT, which is widely used in Kenya.

## Materials and methods

### Ethics statement

The study was approved by the Institutional Review Board of the Kenya Medical Research Institute under protocol numbers KEMRI/SERU 3544. It was conducted in accordance with the ethical standards of the Helsinki Declaration of 1975 as revised in 2000. Written informed consent was obtained by the research assistant using a written form from each study participant. Only participants who were over 18 years were included in this study.

### Study design

This was a cross-sectional prospective study with several method comparisons being performed to evaluate the performance of various sample types tested in the Aptima Assay with the reference plasma VL result in Abbott RT. Venous EDTA blood and finger stick (capillary) blood samples were collected from 3000 consenting study participants. Plasma, venous DBS (VB DBS) and capillary DBS generated by finger stick (FS DBS) were prepared using these samples from each patient. The HIV-1 VL testing was conducted by testing the plasma specimen from each patient in the Abbott RT assay (reference). The VL results in Abbott RT for plasma specimens were used to select patients for testing in Aptima. The plasma and DBS specimens of the first 30–40 patients with Abbott RT VL results of Not Detected, <1.6 and1.6–3 log copies/mL were tested in Aptima. For VL categories above 3 log copies/mL atleast 40 samples were collected for each log of concentration range from 3 to 6 logs. All available samples with HIV VL >6 logs were included in the study. Selection of patients using Abbott RT VL enabled us to assess Aptima performance across the quantitative range of the Aptima for DBS specimens. This also reduced testing burden of having to test 3 sample types in Aptima from over 2500 patients with VL below the assay range of Aptima for DBS specimens.

Method comparisons were performed between the Abbott RT plasma HIV VL result (reference) and VL results of FS DBS, VB DBS and plasma sample types in Aptima. This data was used to assess agreement between VL in plasma tested on Abbott RT and the 3 sample types tested in Aptima at the WHO recommended medical decision point (MDP) of 1000 c/mL [5]. Linear regression and bland Altman analysis were also performed to assess agreement in VL across the assay range for all 3 sample types.

### Study population

The study enrolled a cross-section of consenting HIV positive adults (>18 years) receiving care in 5 medical facilities located in Busia, Alupe, Matayos, Siaya, and Nambale in Kenya, Africa between February and September 2018. Patient information including patient age, gender and ART regimen were collected for all the patients in addition to specimens.

### Sample collection and preparation

Whole blood was collected by venepuncture using BD Vacutainer PPT^TM^ plasma preparation tubes (PPT) from Fisher Scientific while capillary blood was collected by finger-stick. Finger stick DBS were prepared by saturating each of the 5 circles on the Munktell Ahlstrom TFN perforated DBS card with blood. VB DBS were prepared by spotting 70μl of venous blood on each spot on the DBS card for a total of 5 spots per patient. The DBS samples were prepared at the collection site, dried overnight using dry racks (Catalog Number WHA10537173 Sigma-Aldrich, Inc), packaged into Ziploc bags (Catalog Number WHA10548232 Thermo Fisher) with 2 packets of dessicants (Catalog Number WHAWB100003 Sigma-Aldrich, Inc) and shipped to Kenya Medical Research Institute (KEMRI) HIV laboratory in Busia, the next day. The packaged DBS samples were stored at room temperature prior to testing. Whole blood samples were shipped to the laboratory within 6 hours of collection. The whole blood samples were centrifuged at 1,100g for 10 minutes to separate the plasma and stored at -80°C. All samples were de-identified prior to testing in Aptima.

### Laboratory methods

All plasma samples were first tested using the Abbott platform according to the manufacturer’s instructions [26] to generate patient results. The linear quantification range of the Abbott RT assay for plasma sample for 1mL protocol is 40 to 10,000,000 copies/mL(1.60 to 7.00_10_ log copies/mL). Plasma, FS and VB DBS from patients, selected based on their VL in Abbott RT, were tested in the Aptima Assay. For patients with discordant results between plasma VL in Abbott RT and DBS tested in Aptima, the DBS was tested in Abbott RT assay for discordant resolution, if enough number of spots were available. The range of the Abbott RT assay for DBS was 839 to 10,000,000 copies/mL (2.92 to 7.00_10_ log copies/mL).

All the testing for this study was conducted between February and September 2018 by 3 lab technologists in the KEMRI Laboratory in Busia. DBS positive and negative external quality control specimens were included in most of the Aptima runs in this study. Both DBS positive control and negative controls were packed with dessicants and shipped on dry ice from San Diego California to Busia, Kenya and stored at -20°C on receipt at the testing site. The DBS positive control had an assigned concentration of 4.5 log copies per mL.

To determine the performance of Aptima, FS DBS, venous DBS and plasma were tested in Aptima according to manufacturer’s instructions [8, 9].

The Aptima Assay reports quantitative results between 30 and 10,000,000 copies/mL (1.47 to 7.00 log_10_ copies/mL) for plasma samples. For DBS results, a conversion factor in Panther software was used to convert DBS results to copies per mL of HIV-1. The linear quantitative range for Aptima for the DBS sample type is 883 to 10,000,000 copies/mL (2.95 to 7.00_10_ log copies/mL). This assay is run on the fully automated Panther platform. This is a high throughput platform that enables testing up to 320 samples in an 8 hour shift and 560 samples in 12 hours. It enables testing of primary blood collection tubes directly on the platform without the need for aliquoting or manual transfers. Samples can be loaded onto Panther with true random access without the need for batch testing. Multiple assays can be run on Panther at the same time. This platform has a small footprint making it suitable for laboratories of all sizes.

### Data analysis

Only results that met specimen validity criteria in each assay were used for analyses. VL data were transformed into log_10_ copies/mL. Statistical analysis was performed using version 5.30.1 of Analyse-it for Microsoft Excel. SAS JMP version 14.0.0 was also used for data analysis.

The agreement in HIV VL between the different sample types tested in the Aptima assay and the plasma result in Abbott RT were determined at the MDP of 1000 copies/mL (3.0 log copies/mL) using the score method and Kappa statistic was calculated using Analyse-It software. Fisher’s Exact Test was run to assess if the difference in positive and negative agreements seen in plasma and DBS specimens tested in Aptima versus the Abbott RT reference were statistically significant. Patients with Aptima results >3 logs and Abbott RT results <3.0 logs were considered as upward misclassifications by Aptima. Patients with Aptima results <3 logs and Abbott RT results >3.0 logs were considered as downward misclassifications by Aptima. The results of VL in FS DBS in Aptima versus plasma tested in Abbott RT was also trended by patient gender and patient’s ART regimen. For patients with discordant VL results at 1000 copies/mL between the plasma result in Abbott RT and the DBS results in Aptima, the DBS specimens tested in Aptima were tested in Abbott RT if sufficient spots were available for testing.

Only VL results within the quantifiable range of both the Aptima Assay and Abbott RT for the selected sample type was used for Correlation and Bland Altman Analysis. The correlation was determined by simple linear regression with generation of Pearson’s correlation coefficient (*r*). Bland–Altman analysis [27] was used to calculate the average difference between assay results (i.e., bias) and the limit of agreement (LOA) between the assay results. The results of the DBS negative and positive external quality controls were analyzed to assess for contamination and to trend the reproducibility in DBS quantification over the 6 month time frame of the study.

## Results

**Fig 1:**
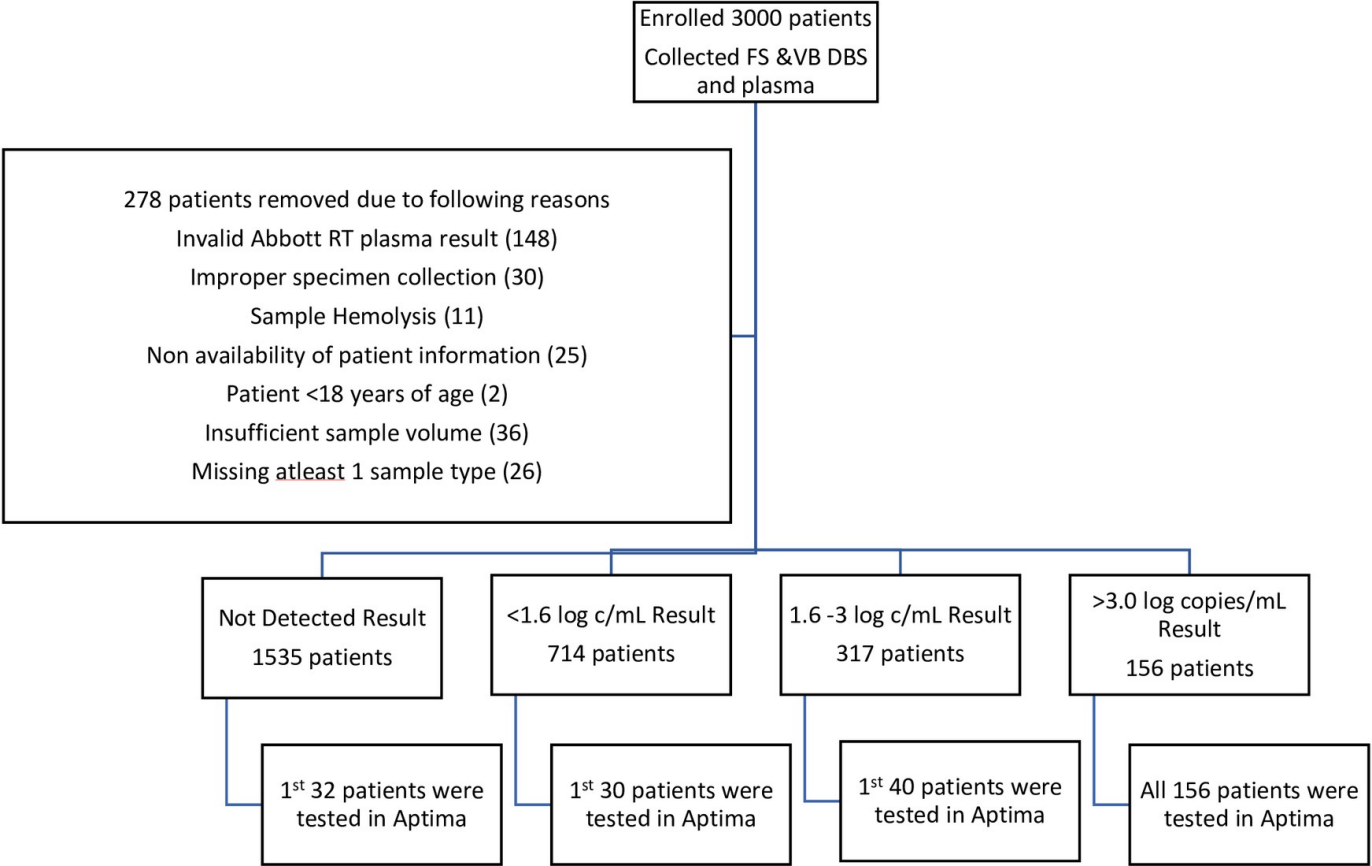
Flow diagram showing inclusion and exclusion criteria for evaluation of performance of the Aptima assay across the quantitative range for DBS specimens.

FS DBS, VB DBS and plasma sample types were collected from the 3000 patients enrolled in this study. The plasma specimens from each patient were tested in Abbott RT. Of these 278 patients were excluded from the study due to the various reasons specified in Fig 1. From the remaining 2722 patients the DBS and plasma from the 1^st^ 30–40 patients with Abbott RT results of “Not detected”, <1.6 log copies/mL and 1.6–3 log copies/mL were tested in Aptima because the VL of these specimens were below the assay range for DBS in Aptima. As seen from Fig 1 all sample types from patients with Abbott RT plasma VL >3 logs were tested in Aptima. This resulted in FS DBS, VB DBS and plasma from 258 patients being tested in Aptima. Table 1 presents the HIV VL distribution of the various sample types for the patients included in this study on being tested in Aptima and Abbott RT assays. Similar levels of HIV detection were observed in FS DBS (90.3%), and VB DBS (91.1%) as that seen on testing plasma specimens in Abbott RT (87.6%) and Aptima (88.0%) with clinical specimens collected from HIV positive patients (Table 1).

**Table 1:** Distribution of HIV VL in the various sample types for the 258 patients included in this study.

HIV Result Category	Abbott RT Plasma Result		Aptima Plasma Result		Aptima FS DBS Result		Aptima VB DBS Result	
Log copies/mL	Not Detected	Detected	Not Detected	Detected	Not Detected	Detected	Not Detected	Detected
Not Detected	32		19	13	12	20	13	19
<1.60	NA	30	9	21	10	20	2	28
1.60–3.00	NA	40	3	37	3	37	7	33
3.01–4.00	NA	62	NA	62	NA	62	1	61
4.01–5.00	NA	53	NA	53	NA	53	NA	53
>5.00	NA	41	NA	41	NA	41	NA	41
**Total N**	**32**	**226**	**31**	**227**	**25**	**233**	**23**	**235**
**% Detected**		**87.6%**	** **	**88.0%**	** **	**90.3%**	** **	**91.1%**

NA = Not Applicable

### Agreement between HIV VL in the 3 sample types tested in Aptima versus the VL in plasma tested in Abbott RT at the clinical medical decision point of 1,000 copies/mL of HIV

More than half these patients (N = 164) had a plasma VL <4 log copies/mL in Abbott RT, making it ideal for evaluation of method agreement at the MDP of 3.0 log copies/mL recommended by WHO (Table 1). Table 2 presents the agreement in HIV VL results between plasma tested in Abbott RT and the 3 sample types tested in Aptima at the MDP of 3.0 log copies/mL. HIV VL results for plasma samples tested in Aptima showed 97.7% total agreement with plasma VL in Abbott RT and had a Kappa statistic of 0.95 (Wald 95% CI 0.91 to 0.99). The positive and negative agreement were 98.1% and 97.1% respectively. Six of the 258 patients (2.3%) had discordant VL results, between plasma tested in Aptima versus Abbott RT, at the MDP of 3.0 log copies /mL. The upward and downward misclassification rate of patients at MDP of 3.0 log copies/mL were 2.9% and 1.9% for plasma results in the two assays. The positive predictive value (PPV) and negative predictive value (NPV) of Aptima at MDP of 3.0 log copies/mL were 98.1% and 97.1% respectively.

**Table 2:** Method agreement of HIV viral load in plasma in Abbott RT compared to that in plasma, fingerstick (FS) DBS and venous(VB) DBS in Aptima at the medical decision point of 1000 copies/mL(3.0 log copies/mL) of HIV recommended by WHO.

					Result	95% LCI	95% UCI
** Aptima plasma**	**Abbott RT plasma**			**Total agreement**	**97.7%**	95.0%	98.9%
	<3.0	>3.0	Total	Positive Agreement/Sensitivity	98.1%	94.5%	99.3%
< 3.0	99 (97.1%)	3 (1.9%)	102 (39.5%)	Negative Agreement/Specificity	97.1%	91.7%	99.0%
> 3.0	3 (2.9%)	153 (98.1%)	156 (60.5%)	Positive Predictive Value	98.1%	94.5%	99.3%
Total	102 (39.5%)	156 (60.5%)	258 (100%)	Negative Predictive Value	97.1%	91.7%	99.0%
**Aptima FS DBS**	**Abbott RT plasma**			**Total agreement**	**92.2%**	88.3%	94.9%
	<3.0	>3.0	Total	Positive Agreement/Sensitivity	92.3%	87.0%	95.5%
< 3,0	94 (92.2%)	12 (7.7%)	106 (41.1%)	Negative Agreement/Specificity	92.2%	85.3%	96.0%
> 3.0	8 (7.8%)	144 (92.3%)	152 (58.9%)	Positive Predictive Value	94.7%	90.0%	97.3%
Total	102 (39.5%)	156 (60.5%)	258 (100%)	Negative Predictive Value	88.7%	81.2%	93.4%
**Aptima VB DBS**	**Abbott RT plasma**			**Total agreement**	**95.3%**	92.0%	97.3%
	<3.0	>3.0	Total	Positive Agreement/Sensitivity	97.4%	93.6%	99.0%
< 3.0	94 (92.2%)	4 (2.6%)	98 (38.0%)	Negative Agreement/Specificity	92.2%	85.3%	96.0%
> 3.0	8 (7.8%)	152 (97.4%)	160 (62.0%)	Positive Predictive Value	95.0%	90.4%	97.4%
Total	102 (39.5%)	156 (60.5%)	258 (100%)	Negative Predictive Value	95.9%	90.0%	98.4%
**Aptima FS DBS**	**Aptima VB DBS**			**Total agreement**	**92.2%**	88.3%	94.9%
	<3.0	>3.0	Total	Positive Agreement/Sensitivity	91.3%	85.9%	94.7%
< 3.0	92 (93.9%)	14 (8.7%)	106 (41.1%)	Negative Agreement/Specificity	93.9%	87.3%	97.2%
> 3.0	6 (6.1%)	146 (91.3%)	152 (58.9%)	Positive Predictive Value	96.1%	91.7%	98.2%
Total	98 (38.0%)	160 (62.0%)	258 (100%)	Negative Predictive Value	86.8%	79.0%	92.0%

This table also shows a method agreement between HIV VL in VB and FS DBS in Aptima.

LCI = Lower Confidence Interval

UCI = Upper Confidence Interval

As seen from Table 2, the total agreement between HIV VL in Aptima VB DBS and Abbott RT plasma at 3.0 log copies/mL was 95.3% with a Kappa statistic of 0.90 (Wald 95% CI 0.85 to 0.96). The positive and negative agreement were 97.4% and 92.2% respectively. Twelve of the 258 patients (4.65%) had discordant results between VB DBS and plasma at the MDP of 3.0 log copies /mL.

The upward and downward misclassification rate of patients based on VB DBS VL at MDP of 3.0 log copies/mL were 7.8% and 2.6%. respectively. Although the upward misclassification rate for Aptima VB DBS (7.8%) was higher than that observed for Aptima plasma, no statistically significant difference was noted between the two on analysis with Fisher’s exact test (p value = 0.21). The PPV and NPV at MDP of 3.0 log copies/mL were 95.0% and 95.9%.

As seen from Table 2, the total agreement between HIV VL in Aptima FS DBS and Abbott RT plasma was 92.2% with a Kappa statistic of 0.84 (Wald 95% CI 0.77 to 0.91). The positive and negative agreement were 92.3 and 92.2% respectively. Twenty of the 258 patients (7.75%) had discordant results between FS DBS and plasma at the MDP of 3.0 log copies /mL. The upward and downward misclassification rate of patients based on FS DBS VL at MDP of 3.0 log copies/mL were 7.8% (8 out of 102 patients with Abbott plasma VL <3.0 log copies/mL) and 7.7%(12 out of 156 patients with Abbott plasma VL >3.0 log copies/mL). There was no statistically significant difference in the number of upward misclassifications between Aptima FS (8) and Aptima plasma (3) compared to plasma VL in Abbott RT (p value = 0.21). However, the difference in number of downward misclassifications between Aptima FS (12) and Aptima plasma (3) compared to plasma VL in Abbott RT was statistically significant on running the Fisher’s exact test (p value = 0.03). The positive predictive value (PPV) and negative predictive value (NPV) for FS DBS at MDP of 3.0 log copies/mL were 94.7% and 88.7% respectively.

FS DBS and VB DBS VL results in Aptima had a total agreement of 92.2% in VL results at the MDP with positive and negative agreements of 91.3% and 93.9% respectively (Table 2).

### Discordant resolution for discordant VL results at MDP of 1000 c/mL between plasma tested in Abbott RT and DBS tested in Aptima

Table 3 presents the HIV VL results for all 3 sample types for patients who had discordant results between the plasma result in Abbott RT and FS and VB DBS in Aptima at the medical decision point of 3.0 log copies/mL. As seen from Table 3 all discordant samples had VL < 3.48 log copies/mL (<3000 copies/mL) on testing plasma in both Abbott RT and Aptima Assays.

**Table 3:** HIV VL results from patients who had discordant results between VL plasma tested in Abbott RT compared to VL in FS and VB DBS tested in Aptima at the medical decision point of 3 log copies/mL of HIV.

Number	Result Category	Aptima Plasma	Abbott Plasma	Aptima FS DBS	Aptima VB DBS	Abbott FS DBS	Abbott VB DBS
1	**Abbott Plasma** VL >3 log copies/mL and **Aptima FS DBS** <3 log copies/mL	3.47	**3.33**	**<2.95**	3.03	Not Detected	2.95
2		3.39	**3.12**	**<2.95**	3.36	Not Detected	<2.92
3		3.36	**3.11**	**2.98**	3.15	<2.92	<2.92
4		3.31	**3.48**	**<2.95**	3.39	Not Detected	3.40
5		3.16	**3.12**	**<2.95**	3.03	Not Detected	<2.92
6		3.13	**3.29**	**<2.95**	3.50	Not Detected	Not Detected
7*		3.12	**3.07**	**<2.95**	<2.95	Not Detected	<2.92
8*		3.11	**3.03**	**<2.95**	<2.95	Not Detected	Not Detected
9		3.07	**3.10**	**2.96**	3.39	<2.92	Not Detected
10*^		2.86	**3.03**	**2.99**	<2.95	Not Detected	<2.92
11^		2.65	**3.05**	**2.98**	3.14	Not Available	Not Available
12^		2.18	**3.07**	**<2.95**	3.02	Not Available	Not Available
13^	**Abbott Plasma** VL <3 log copies/mL and **Aptima FS DBS** >3 log copies/mL	3.01	**2.88**	**3.13**	<2.95	<2.92	<2.92
14*		2.95	**2.38**	**3.30**	3.23	Not Available	Not Available
15		2.21	**<1.60**	**3.07**	<2.95	Not Available	Not Available
16		1.89	**1.95**	**3.15**	<2.95	Not Available	Not Available
17*		1.85	**2.06**	**3.27**	3.10	Not Available	Not Available
18		<1.47	**1.76**	**3.15**	2.96	Not Available	Not Available
19*		<1.47	**1.77**	**3.12**	3.28	Not Available	Not Available
20		<1.47	**<1.60**	**3.20**	<2.95	Not Available	Not Available
1*	**Abbott Plasma** VL>3 log copies/mL and **Aptima VB DBS** <3 log copies/mL	3.12	**3.07**	<2.95	**<2.95**	Not Detected	<2.92
2*		3.11	**3.03**	<2.95	**<2.95**	Not Detected	Not Detected
3		3.62	**3.45**	4.03	**Not Detected**	3.01	Not Detected
4*		2.86	**3.03**	2.99	**<2.95**	Not Available	Not Available
5*	**Abbott Plasma** VL<3 log copies/mL and **Aptima VB DBS** > 3 log copies/ml	2.95	**2.38**	3.30	**3.23**	Not Available	Not Available
6		Not Detected	**<1.60**	<2.95	**3.03**	Not Available	Not Available
7		2.52	**2.61**	<2.95	**3.09**	Not Available	Not Available
8*		1.85	**2.06**	3.27	**3.10**	Not Available	Not Available
9*		<1.47	**1.77**	3.12	**3.28**	Not Available	Not Available
10		1.69	**1.76**	<2.95	**3.17**	Not Detected	<2.92
11		2.00	**2.10**	<2.95	**3.39**	Not Detected	Not Detected
12		<1.47	**Not Detected**	Not Detected	**3.43**	Not Detected	<2.92

The 6 patients marked with “*” were discordants for both FS and VB DBS tested in Aptima in the comparison to Abbott plasma VL. The 4 patients marked with “^” also had discordant results for plasma tested in Abbott RT and Aptima.

Table 3 also includes the results from testing the same DBS specimens in Abbott RT assay, whenever specimens were available. Interestingly all patients with <3 log copies/mL VL in FS and VB DBS in Aptima versus >3.0 log copies/mL in Abbott RT plasma also had <3.0 log copies/mL or “Not Detected” results on testing the same DBS specimens in Abbott RT.

All 12 patients with Abbott RT plasma VL>3.0 and Aptima FS DBS VL<3.0 also had VL results <3 log copies/mL on testing the FS DBS in Abbott RT. Nine of these had VL results >3 log copies/mL for Aptima plasma and VB DBS making the FS DBS lower than that seen in other sample types. For the 8 patients with Abbott RT plasma VL<3.0 and FS DBS VL >3 log copies/mL, 7 had VL <3 log copies/mL in Aptima plasma and 5 had VB DBS VL <3 log copies/mL.

Six patients in Table 3 had discordant VL results not only for FS DBS but also VB DBS in Aptima compared to the Abbott RT plasma result. For 3 of these patients the VL in FS and VB DBS specimens tested in both Aptima and Abbott RT were just below <3 log copies/mL with VL in plasma tested in Abbott RT being just >3 log copies/mL (ie 3.03 to 3.10 log copies/mL). For these 3 patients the discordant results between plasma and DBS is likely to be an artefact of partitioning VL results at the MDP of 3 log copies/mL even though they have similar HIV concentrations. For the remaining 3 patients with FS and VB DBS VL >3 log copies/mL and plasma VL in both assays <3 log copies/mL degradation of HIV RNA in the plasma specimens prior to testing cannot be ruled out.

The VL of 4 of the 6 patients with discordant results between plasma tested in Aptima and Abbott RT is also shown in Table 3. The 5^th^ patient had Abbott plasma VL of 1.98 log copies/mL while the VL results in Aptima for plasma was 3.13 log copies/mL with both DBS specimens having <2.95 detected results. The 6^th^ patient had a discordant result possibly due to contamination of the plasma sample in Aptima which reported “Not Detected” on retest in Aptima. This sample had a “Not Detected” result for plasma in Abbott RT and <2.95 detected results for the FS and VB DBS tested in Aptima. Therefore the plasma VL results in Aptima and Abbott RT showed good agreement for atleast 5 of these patients with all the quantifiable results being within 0.5 log of each other although they were discordant at the MDP of 3.0 log copies/mL.

### Method agreement between HIV VL in Abbott RT and Aptima FS DBS analysed by patient gender and ART regimen

Among the 258 patients included in this study, 178 were females and 80 were males (Table 4). A total of 217 patients were enrolled in 1^st^ line ART regimens [5, 6] 215 being treated with 3 drugs ie lamivudine (3TC) in combination with either Zidovudine (AZT) or Tenofovir (TDF) and either Nevirapine (NVP) or Efavirenz (EFV). Two patients were on a first line regimen containing TDF, 3TC and Dolutegravir (DTG). Twenty-eight patients were on second line ART regimens [5] containing combinations of Lopinavir / Ritonavir (LPV/r) or Atazanavir /Ritonavir (ATV/r). Information on ART regimen was not available for 13 patients. Nineteen of the 20 discordant results were seen in the 217 of patients who were on 1^st^ line ART regimens [5] with no trend based on patient gender. One discordant result was seen in a patient whose treatment regimen was not known.

**Table 4:** Method agreement of HIV viral load in plasma in Abbott RT compared to in fingerstick DBS in Aptima at the medical decision point of 1000 copies/mL of HIV, sorted by patient gender and treatment regimen.

	Abbott RT Plasma<3.0 Aptima FS DBS<3.0 log copies/mL		Abbott RT Plasma<3.0 Aptima FS DBS>3.0 log copies/mL		Abbott RT Plasma>3.0 Aptima FS DBS<3.0 log copies/mL		Abbott RT Plasma>3.0 Aptima FS DBS>3.0 log copies/mL		Grand Total
Row Labels	Female	Male	Female	Male	Female	Male	Female	Male	
AZT+3TC+EFV*	3	None	None	1	3	None	8	2	17
AZT+3TC+NVP*	21	7	1	1	None	1	11	7	49
AZT+3TC+ATV/r^	3	None	None	None	None	None	5	2	10
AZT+3TC+LPV/r^	None	None	None	None	None	None	1	2	3
Other	1	3	1	None	None	None	7	1	13
TDF+3TC+ATV/r^	5	None	None	None	None	None	4	6	15
TDF+3TC+DTG*	None	None	None	None	None	None	1	1	2
TDF+3TC+EFV*	28	14	1	2	3	2	54	22	126
TDF+3TC+NVP*	8	1	1	None	1	2	7	3	23
**Grand Total**	**69**	**25**	**4**	**4**	**7**	**5**	**98**	**46**	**258**

Regimens marked with”*” and “^” are 1^st^ and 2^nd^ line ART regimens recommended by WHO [6].

### Comparison of viral load measurement across the assay range and Bland Altman analysis

One hundred and seventy-six patients had quantifiable results for plasma in both Abbott RT and Aptima with a median viral load of 4.34 log copies/mL in Aptima. Only 4 samples with quantifiable result in both assays had a difference in VL of >1 log with the maximum difference being 1.15 log copies. In all 4 cases Aptima had higher VL than Abbott RT. Correlation of VL results between plasma samples tested in Abbott and Aptima is not shown because there are several publications [10–20] demonstrating excellent agreement between plasma VL results in both these assays across the assay range.

**Fig 2:**
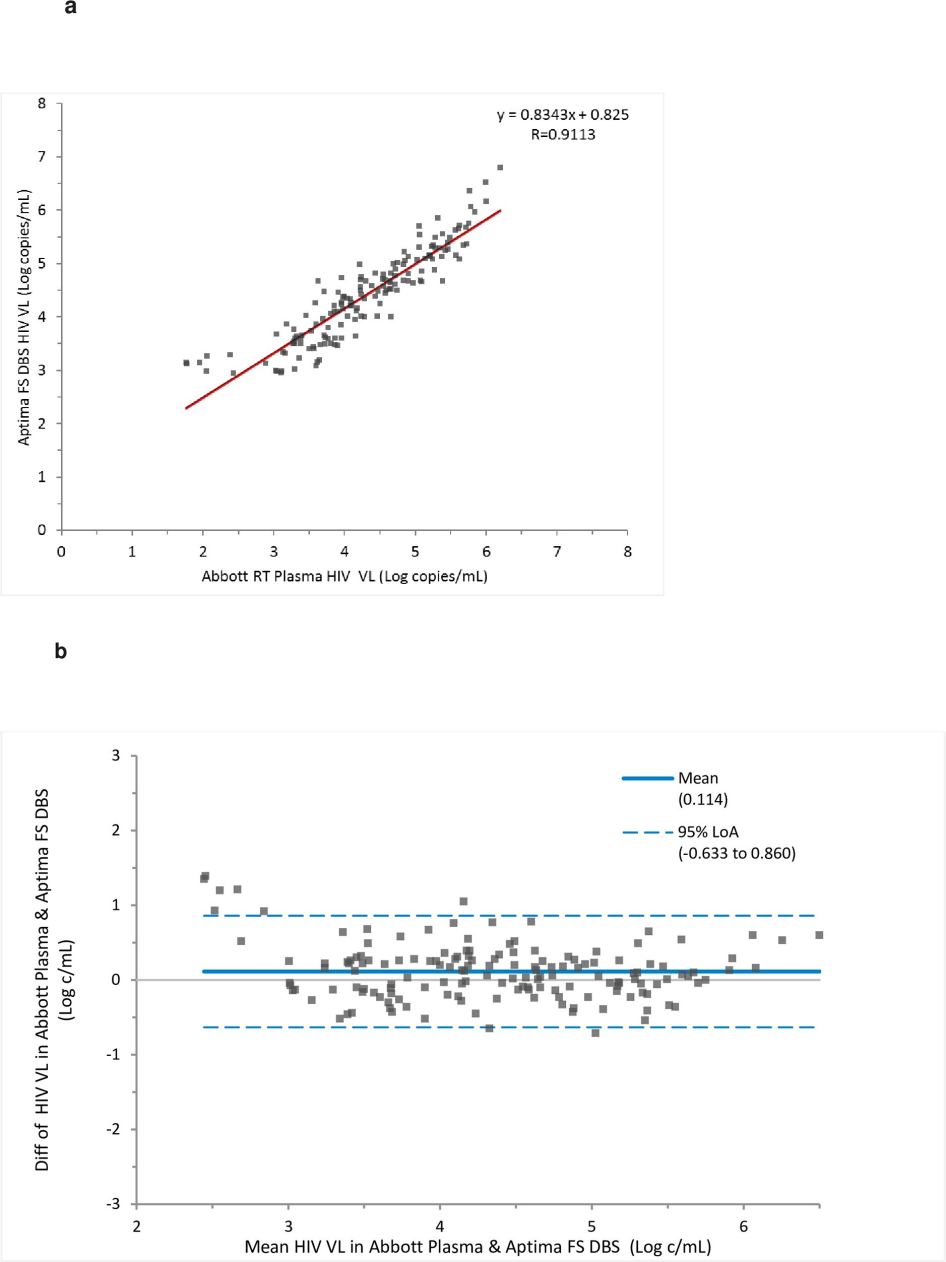
**a.** Comparison of HIV VL in FS DBS tested in Aptima versus plasma tested in Abbott RT across the assay range. **b.** Bland Altman analysis of the difference in HIV viral load between FS DBS in Aptima and plasma tested in Abbott RT assay.

Samples from 156 patients had quantifiable results for the comparison of viral load between Abbott RT and Aptima for plasma and fingerstick DBS respectively. As seen from Fig 2A the linear regression gave a Pearson’s correlation coefficient (*r*) of 0.91. Bland-Altman analysis of the difference in Aptima FS DBS versus Abbott plasma VL results relative to the average values is shown in Fig 2B. The mean bias was 0.114 log copies/mL with 95% CI of limit of agreement being -0.63 to 0.86. for the comparison between plasma results in Abbott RT and FS DBS results in Aptima.

**Fig 3:**
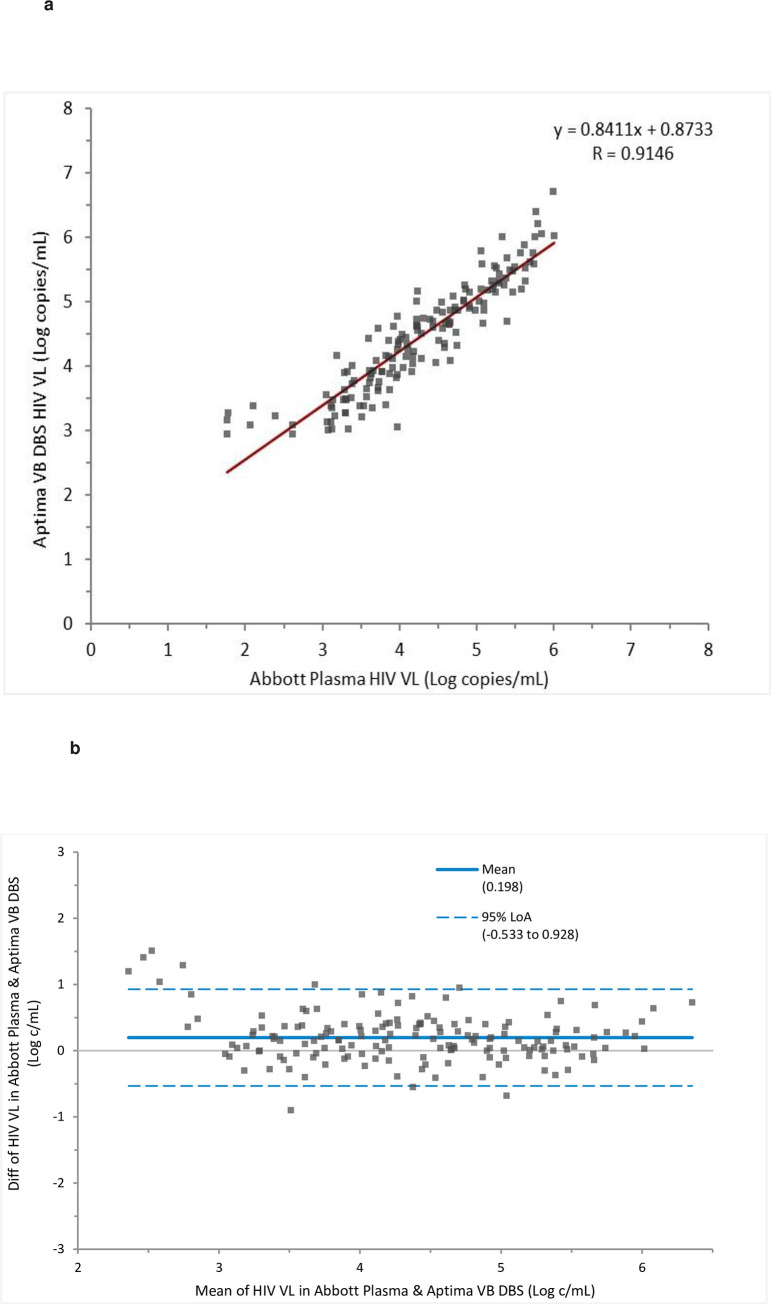
**a.** Comparison of HIV VL in VB DBS tested in Aptima versus plasma tested in Abbott RT across the assay range. **b.** Bland Altman analysis of the difference in HIV viral load between VB DBS Aptima and plasma tested in Abbott RT assay.

A total of 159 samples were included in the linear regression analysis between HIV VL of plasma samples tested in Abbott RT and VB DBS tested in Aptima because they had quantifiable results for both sample types. The analysis gave a Pearson’s correlation coefficient (*r*) of 0.91 (Fig 3A). Bland-Altman analysis gave a bias of 0.198 with limits of agreement ranging from -0.533 to 0.928 (Fig 3B).

### DBS external quality control results

HIV VL recovery of 1 replicate of the external Quality control DBS was trended over 35 Aptima runs performed between February and August 2018 by atleast 3 different technologists. All replicates of the DBS negative controls reported “Not Detected Results in all the runs. The average recovery of the DBS positive control was 4.40 log copies/mL (95% CI 4.06 to 4.74 log copies/mL) versus the assigned concentration of 4.50 log copies/mL assigned by the manufacturer. The standard deviation log copy was 0.11 for the results of this specimen tested over a 6 month time frame.

## Discussion

Meticulous clinical evaluations of new HIV VL assays run on high throughput automated platforms are required to identify assays that will enable scale up of HIV VL monitoring, to meet UNAIDS goals to control the HIV epidemic [1]. These assays need to quantify HIV VL not only from plasma but also DBS because testing FS DBS is crucial to enable VL monitoring of patients in rural and resource limited settings. Currently there is no published information available comparing performance the FS DBS testing in Aptima with paired plasma result at the medical decision point of 1000 copies/mL.

The Aptima Assay has CE IVD approval for HIV diagnosis and VL monitoring using both plasma and DBS specimens and is run on the high throughput fully automated Panther platform. Various investigators have demonstrated the equivalency of HIV VL results generated in Aptima with assays ranging from Abbott RT to Roche CAP/CTM using the gold standard plasma sample type [10–20] and VB DBS [23–25]. This is the first study to evaluate the performance of the Aptima Assay with FS DBS. In this study we compared the HIV VL in plasma, FS and VB DBS specimens in the Aptima Assay to the plasma VL in Abbott RT.

All the clinical specimens tested in this study were collected from HIV positive patients who received care at 5 different medical facilities in rural Kenya.

Although DBS has been shown to be less sensitive than plasma in analytical studies [8, 9, 26] similar levels of HIV detection were observed in FS DBS (90.3%), and VB DBS(91.1%) as that seen on testing plasma specimens in Abbott RT (87.6%) and Aptima (88.0%) as shown in Table 1. The high level of detection seen in DBS specimens from HIV positive patients, making them comparable to plasma suggest that DBS testing in Aptima may be a viable alternative to testing plasma for diagnosis of HIV infection.

The results of this study show excellent agreement (97.7%) in VL results between Abbott RT and the Aptima Assay at the MDP 3.0 log copies/mL on testing plasma, which is the gold standard sample type for HIV VL monitoring. The upward and downward misclassification of VL results on testing plasma in Aptima versus Abbott RT was low at 2.9 and 1.9% (Table 2).

The total agreement of 95.3% between Aptima VB DBS VL and Abbott RT plasma result was not significantly different from the total agreement (97.7%) in VL of the plasma specimens tested in both assays in this study (Table 2). A similar level of total agreement (94.7%) of results was reported by Sahoo et al [25] on comparing VL in VB DBS and plasma in Aptima. The positive agreement of 97.4% and the percentage of patients with downward misclassification (2.9%) for VB DBS VL in Aptima were also similar to that seen with Aptima plasma. The positive predictive value (PPV) and negative predictive value (NPV) as well as their lower 95% CI were >90% for both VB DBS and plasma tested in Aptima compared to the Abbott RT plasma reference result. The Kappa statistic for comparisons between plasma tested in both assays and Aptima VB DBS versus Abbott RT plasma were 0.95 and 0.90 respectively indicating excellent agreement for both these comparisons. These results indicate that VB DBS VL in Aptima was equivalent to plasma results in Aptima and Abbott RT.

VL results of FS DBS also showed good total agreement (92.2%) with Abbott RT VL with positive and negative agreement of 92.3% and 92.2% respectively and kappa statistic of 0.84. However, the positive and negative agreement as well as PPV [94.7%] and NPV [88.7%] for VL results for FS DBS tested in Aptima were slightly lower than that seen for the comparison between plasma results in Aptima and Abbott RT.

The positive and negative agreement of Aptima FS and VB DBS VL compared to Abbott RT VL observed in this study was equivalent or better than those reported by other investigators for comparisons of DBS and plasma VL results in Abbott RT assays and NucliSENS Easy-Q HIV-1 version 2.0 assays [28–33]. The negative agreement (92.2%) between FS and VB VL versus Abbott plasma VL seen in this study was significantly better than the 17% negative agreement reported for testing DBS in Roche CAP/CTM at MDP of 3.0 log copies/mL [28]. The Kappa statistic for total agreement for all 3 sample types (0.95, 0.90, 0.84) tested in Aptima was also significantly better than the Kappa statistic (0.2) reported by Schmitz et al [29] for comparison of DBS VL in CAP/CTM versus Abbott RT plasma VL. The reason for the high negative agreement of DBS results in Aptima compared to CAP/CTM is unclear but one potential cause could be the isothermal transcription mediated amplification technology used by Aptima which does not effectively amplify double stranded DNA [8, 9]. Other investigators have reported high negative agreement for DBS tested with assays that use technologies that do not effectively amplify HIV proviral DNA [32, 33].

The HIV VL of 8 patients with Abbott RT results <3 log copies/mL were misclassified upward (>3 log copies/mL) by FS and VB DBS tested in Aptima resulting in an upward misclassification rate of 7.8%. This rate of upward misclassification is not significantly different from the 2.2–6.9% than that has been reported by other investigators when testing DBS in Abbott RT and NucliSENS Easy-Q HIV-1 version 2.0 assays [29, 30, 32] The upward misclassification rate for Aptima was much better than the 69% reported for CAP/CTM [29]. The risk to patient health for the patients with these discordant results> 3 log copies/mL should be low because the WHO guideline [5] as well as current Kenyan Medical guideline [6] recommends confirmation of VL before changing the ART regimen. Even if the patient’s treatment regimen is changed based on these VL results they should still be effectively treated for HIV and risk of transmission should be low. Therefore these discordant results with upward misclassification should have minimal public health impact.

Downward misclassifications based on DBS results compared to plasma have more clinical and public health relevance because these DBS results could cause patients to be misclassified as virally suppressed when they may be failing ART and developing drug resistance. Only 4 patients (2.6%) had downward misclassification, with VB DBS VL in Aptima being <3 log copies/mL versus Abbott plasma VL being >3 log copies/mL. A total of 12 patients had downward misclassification of results based on Aptima FS DBS VL being <3 log copies/mL versus Abbott RT plasma VL being >3.0 log copies/mL. The 7.7% (ie 12 out of 156 patients with plasma VL >3 log copies/mL as shown in Table 2) downward misclassification of patients based on FS DBS VL results in Aptima is lower than the 11.9–20.0% that reported by other investigators for FS DBS at the MDP of 3.0 log copies/mL for DBS tested in Abbott RT and NucliSENS Easy-Q HIV-1 version 2.0 assays [28, 30, 32].

The plasma VL was between 3 and 3.47 log copies/mL in both Abbott RT and Aptima for all the patients with downward misclassifications based on FS and VB DBS VL Results. (Table 3). For the 12 patients who were misclassified based on FS DBS results 9 of these also had VB DBS VL in this same concentration range as plasma on testing in Aptima. These results suggest that atleast some of these differences in VL in FS DBS could be due to differences in preparation of FS DBS under field conditions while others could be due to variability in VL quantitation by testing just one replicate of each specimen to assess agreement at the MDP of 3.0 log copies/mL [34]. Several investigators have reported a high rate of discordant rates between DBS and plasma VL in different assays at the MDP of 3.0 log copies/mL when the plasma VL of the specimens are less than 3.5–3.7 log copies/mL ie 3000–5000 copies/mL [28–30, 32, 33]. Although other investigators have reported associations between patient gender and ART regimen with successful viral load suppression, no trends were observed in this study [35].

The strength of this study was that it was a prospective evaluation of paired plasma, FS and VB DBS specimens, collected from patients across 5 different medical facilities in Kenya by the personnel at those sites. Also, more than half the patients in this study had VL <4.0 log copies/mL making this study ideal for the evaluation of accuracy of plasma, FS and VB DBS at MDP of 3.0 log copies/mL.

One limitation of this study was the inability to perform discordant testing using Abbott RT for some DBS specimens with discordant results. Also, HIV subtyping was not performed for specimens in this study. However, many studies have demonstrated equivalent subtype quantification in Aptima [11–20]. At least two of these studies [17, 20] tested many subtype samples collected from around the world including Africa. These studies include the predominant HIV subtypes seen in Kenya such as subtype A, D, C, G and recombinants [21, 22, 36, 37]. The good agreement seen between Aptima VL results and Abbott RT seen in this study also suggest that subtype quantification of Aptima is equivalent to that of Abbott RT for the HIV subtypes prevalent in Kenya.

The results of this study show that that plasma, FS DBS and VB DBS could be used interchangeably to determine HIV VL for assessment of treatment failure at the MDP of 1000 c/mL (3.0 log copies/mL). Monitoring VL using DBS specimens in Aptima is good option for clinical management of HIV patients especially under resource limited conditions where phlebotomy, timely processing and shipping of plasma samples under refrigerated conditions is difficult. The high level of agreement between plasma HIV VL in the Abbott RT assay and Aptima implies that the Aptima Assay can be used interchangeably with Abbott Real-Time assay for HIV viral load determination in plasma and DBS. The positive and negative agreement for DBS tested in Aptima compared to Abbott plasma VL meet the WHO recommendations for performance for viral load monitoring [31].

The Panther Platform has a small footprint, complete automation and high throughput [18]. In our hands, Aptima returned total agreement >90% for all 3 sample types compared to the Abbott RT reference result. We observed some operational advantages of the Aptima assay in the laboratory workflow during the study. Aptima is run on the Panther instrument platform that is fully automated and allows random and continuous loading of test samples. It allows processing of up to 500–700 tests a day, returning results for each sample in about 2.5-hours. This enables high flexibility to adapt to low or high-throughput testing. The ability to load primary tubes of plasma samples and lack of multiple transfer steps during plasma and DBS processing also improves sample traceability.

## Conclusions

In conclusion, the Aptima HIV-1 Quant Dx assay has performance characteristics that are comparable with those of the Abbott Real-Time assay for VL monitoring using both plasma and DBS samples. The clinical performance of the Aptima Assay as well as its improved sample traceability, high throughput, complete automation, ease of use and small footprint of the Panther system makes it an attractive solution for routine monitoring of HIV-1 VL in clinical laboratories, especially those with increased demand for viral load testing and infrastructural challenges such as laboratory space.

## References

[1] 90-90-90: an ambitious treatment target to help end the AIDS epidemic.2017 Joint United Nations Programme on HIV/AIDS, Geneva, Switzerland. Available from https://www.unaids.org/en/resources/documents/2017/90-90-90.

[2] UNAIDS. Global AIDs Update (2019) Joint United Nations Programme on HIV/AIDS, Geneva, Switzerland. Available from: https://www.unaids.org/en/resources/documents/2019/2019-global-AIDS-update

[3] Rutherford GW, Anglemyer A, Easterbrook PJ, Horvath T, Vitoria M, Penazzato M, et al. Predicting treatment failure in adults and children on antiretroviral therapy: a systematic review of the performance characteristics of the 2010 WHO immunologic and clinical criteria for virologic failure. AIDS. 2014;28 Suppl 2:S161–9. 10.1097/QAD.0000000000000236 . 24849476

[4] Barth RE, van der Loeff MF, Schuurman R, Hoepelman AI, Wensing AM. Virological follow-up of adult patients in antiretroviral treatment programmes in sub-Saharan Africa: a systematic review. Lancet Infect Dis. 2010;10(3):155–66. 10.1016/S1473-3099(09)70328-7 . 20185094

[5] Consolidated guidelines on the use of antiretroviral drugs for treating and preventing HIV infection. World Health Organization, Geneva, Switzerland. 2016. Available from: https://www.who.int/hiv/pub/arv/arv-2016/en/27466667

[6] Guidelines on Use of Antiretroviral Drugs for Treating and Preventing HIV Infections in Kenya 2018 Edition. Kenya Ministry of Health. Available from: https://www.nascop.or.ke/new-guidelines/

[7] Sloma CR, Germer JJ, Gerads TM, Mandrekar JN, Mitchell PS, Yao JD. Comparison of the Abbott RealTime Human Immunodeficiency Virus Type-1 (HIV-1) Assay to the Cobas AmpliPrep/Cobas TaqMan HIV-1 Test: Workflow, Reliability and Direct Costs. J. clin. Microbiol. 2009;47: 889–895 10.1128/JCM.02231-08 19193837 PMC2668302

[8] Package insert for Aptima HIV-1 Quant Dx assay. Available from: https://www.hologic.com/package-inserts/diagnostic-products/aptima-hiv-1-quant-dx-assay-ce-ivd

[9] Package Insert for Dried Blood Spot supplement to the Aptima HIV-1 quant Dx assay. 2019. Available from: https://www.hologic.com/package-inserts/diagnostic-products/aptima-hiv-1-quant-dx-

[10] Saune K, Raymond S, Boineau J, Pasquier C, Izopet J. Detection and quantification of HIV-1 RNA with a fully automated transcription-mediated-amplification assay. J Clin Virol. 2016;84:70–3. 10.1016/j.jcv.2016.09.002 . 27728849

[11] Amendola A, Pisciotta M, Aleo L, Ferraioli V, Angeletti C, Capobianchi MR. Evaluation of the Aptima HIV-1 Quant Dx assay for HIV-1 RNA viral load detection and quantitation in plasma of HIV-1-infected individuals: A comparison with Abbott RealTime HIV-1 assay. J Med Virol. 2016;88(9):1535–44. 10.1002/jmv.24493 . 26864171 PMC6585778

[12] Hatzakis A, Papachristou H, Nair SJ, Fortunko J, Foote T, Kim H, et al. Analytical characteristics and comparative evaluation of Aptima HIV-1 Quant Dx assay with Ampliprep/COBAS TaqMan HIV-1 test v2.0. Virol J. 2016;13(1):176–84. 10.1186/s12985-016-0627-y 27769309 PMC5073876

[13] Hopkins M, Hau S, Tiernan C, Papadimitropoulos A, Chawla A, Beloukas A, et al. Comparative performance of the new Aptima HIV-1 Quant Dx assay with three commercial PCR-based HIV-1 RNA quantitation assays. J Clin Virol. 2015;69:56–62. 10.1016/j.jcv.2015.05.020 . 26209380

[14] Mor O, Gozlan Y, Wax M, Mileguir F, Rakovsky A, Noy B, et al. Evaluation of the RealTime HIV-1, Xpert HIV-1, and Aptima HIV-1 Quant Dx Assays in Comparison to the NucliSens EasyQ HIV-1 v2.0 Assay for Quantification of HIV-1 Viral Load. J Clin Microbiol. 2015;53(11):3458–65. 10.1128/JCM.01806-15 26292298 PMC4609691

[15] Schalasta G, Borner A, Speicher A, Enders M. Comparative evaluation of the Aptima HIV-1 Quant Dx assay and COBAS TaqMan HIV-1 v2.0 assay using the Roche High Pure System for the quantification of HIV-1 RNA in plasma. Clin Chem Lab Med. 2016;54(3):493–9. 10.1515/cclm-2015-0522 . 26351942

[16] Schonning K, Johansen K, Landt B, Benfield T, Westh H. Comparison of the Hologic Aptima HIV-1 Quant Dx Assay to the Roche COBAS Ampliprep/COBAS TaqMan HIV-1 Test v2.0 for the quantification of HIV-1 RNA in plasma samples. J Clin Virol. 2017;92:14–9. 10.1016/j.jcv.2017.05.006 . 28505569

[17] Manak MM, Hack HR, Nair SV, Worlock A, Malia JA, Peel SA, et al. Evaluation of Hologic Aptima HIV-1 Quant Dx Assay on the Panther System on HIV Subtypes. J Clin Microbiol. 2016;54(10):2575–81. 10.1128/JCM.01350-16 27510829 PMC5035401

[18] Sam SS, Kurpewski JR, Cu-Uvin S, Caliendo AM. Evaluation of Performance Characteristics of the Aptima HIV-1 Quant Dx Assay for Detection and Quantitation of Human Immunodeficiency Virus Type 1 in Plasma and Cervicovaginal Lavage Samples. J Clin Microbiol. 2016;54(4):1036–41. 10.1128/JCM.03289-15 26842702 PMC4809915

[19] Nair SV, Kim HC, Fortunko J, Foote T, Peling T, Tran C, et al. Aptima HIV-1 Quant Dx—A fully automated assay for both diagnosis and quantification of HIV-1. J Clin Virol. 2016;77:46–54. 10.1016/j.jcv.2016.02.002 . 26896710

[20] Wiesmann F, Ehret R, Naeth G, Daumer M, Fuhrmann J, Kaiser R, et al Multicenter Evaluation of Two Next Generation HIV Quantitative Assays, Aptima Quant Dx and Cobas 6800 in Comparison to the RealTime HIV-1 Reference Assay. J. Clin Microbiol 208: 56 (10) e00292–18 10.1128/JCM.00292-18 PMC6156314 30068537

[21] Bbosa N, Kaleebu P, and Ssemwanga D. HIV subtype diversity worldwide. Current Opinion in HIV and AIDS: 2019; 14(3):153–160 10.1097/COH.0000000000000534 30882484

[22] Gounder K, Oyaro M, Padayachi N, Zulu TM, De Oliveira T, Wylie J, et al. Complex Subtype Diversity of HIV-1 Among Drug Users in Major Kenyan Cities. AIDS Res Hum Retroviruses 2017; 33(5);500–510 10.1089/AID.2016.0321 28068781 PMC5439455

[23] Yek C, Massanella M, Peling T, Lednovich K, Nair SV, Worlock A, et al. Evaluation of the Aptima HIV-1 Quant Dx Assay for HIV-1 RNA Quantitation in Different Biological Specimen Types. J Clin Microbiol. 2017;55(8):2544–53. 10.1128/JCM.00425-17 28592548 PMC5527433

[24] Carrera A, Sherring J, McNally L, Lowe P, Cunningham PH. Performance evaluation of the aptima hiv-1 quant dx assay for detection of hiv-1 in plasma and dried blood spots (dbs) Sexually Transmitted Infections. 2015: 91(2):A1–258 Available from: https://sti.bmj.com/content/91/Suppl_2/A211.1

[25] Sahoo MK, Varghese V, White E, Winslow M, Katzenstein DA, Shafer RW, et al. Evaluation of the Aptima HIV-1 Quant Dx Assay Using Plasma and Dried Blood Spots. J Clin Microbiol. 2016;54(10):2597–601. 10.1128/JCM.01569-16 27535684 PMC5035416

[26] Abbott RealTime HIV-1 Package Insert for plasma and DBS quantification Available from: https://www.molecular.abbott/us/en/products/infectious-disease/realtime-hiv-1-viral-load

[27] Bland JM, Altman DG. Statistical methods for assessing agreement between two methods of clinical measurement. Lancet. 1986;1(8476):307–10. . 2868172

[28] Zeh C, Ndiege K, Inzaule S, Achieng R, Williamson J, Chang JC, et al. Evaluation of the performance of Abbott m2000 and Roche COBAS Ampliprep/COBAS Taqman assays for HIV-1 viral load determination using dried blood spots and dried plasma spots in Kenya. 2017 6 16;12(6):e0179316 PLoS One 10.1371/journal.pone.0179316 PMCID: PMC5473550 28622370 PMC5473550

[29] Schmitz ME, Agolory S, Junghae M, Broyles LN, Kimeu M, Ombayao J et al. Field Evaluation of Dried Blood Spots for HIV Viral Load Monitoring in Adults and Children Receiving Antiretroviral Treatment in Kenya: Implications for Scale-up in Resource Limited Settings. J Acquir Immune Defic Syndr. 2017 4 1;74(4):399–406. 10.1097/QAI.0000000000001275 28002185

[30] Chang J, Sousa A, Sabatier J, Assane M, Zhang G, Bila D, et al Performance characteristics of finger-stick dried blood spots (DBS) on the determination of human immunodeficiency virus (HIV) treatment failure in a pediatric population in Mozambique PLoS One 2017 7 13;12(7):e0181054. 10.1371/journal.pone.0181054 PMCID: PMC5509298 28704560 PMC5509298

[31] Technical and Operational Considerations for Implementing HIV Viral Load Testing 2014 World Health Organization, Geneva, Switzerland. Available from: https://www.who.int/hiv/pub/arv/viral-load-testing-technical-update/en/

[32] Mavedzenge SN, Davey C, Chirenje T, Mushati P, Mtetwa S, Dirawo J, et al. Cowan FC Finger Prick Dried Blood Spots for HIV Viral Load Measurement in Field Conditions in Zimbabwe PLoS One. 2015 5 22;10(5):e0126878 10.1371/journal.pone.0126878 PMCID: PMC4441418 26001044 PMC4441418

[33] Fajardo E., Metcalf C.A., Chaillet P., Aleixo L., Pannus P., Panunzi I, et al. Prospective evaluation of diagnostic accuracy of dried blood spots from finger prick samples for determination of HIV-1 load with the NucliSENS Easy-Q HIV-1 version 2.0 assay in Malawi. J Clin Microbiol. 2014; 52: 1343±1351 PPubMed MID: 10.1128/JCM.03519-13 24501032 PMC3993687

[34] Sollis KA, Smit PW, Fiscus S, Ford N, Vitoria M, Essajee S, et al Systematic Review of the Performance of HIV Viral Load Technologies on Plasma Samples PLoS One. 2014; 9(2): e85869. 10.1371/journal.pone.0085869 24558359 PMC3928047

[35] Darcis G, Maes N, Pasternak AO, Sauvage A, Frippiat F, Meuris C et al. Detectability of HIV residual viremia despite therapy is highly associated with treatment with a protease inhibitor-based combination antiretroviral therapy Antimicrob. Agents Chemother. 2 2020, 64 (3) e01902–19; 10.1128/AAC.01902-19 31818822 PMC7038286

[36] Kiptoo M, Brooks J, Lihana RW, Sandstrom S, Ng’ang’a Z, Kinyua J et al. HIV-1 drug resistance-associated mutations among HIV-1 infected drug-naïve antenatal clinic attendees in rural Kenya. *BMC Infect Dis*. 2013;13:517–20. 10.1186/1471-2334-13-517 PMCID: PMC4228423 24180455 PMC4228423

[37] Onsongo S, Abidi SH, Khamadi S, Shah R, Kageha S, Ojwang P et al. Prevalence of Transmitted Drug Resistance Mutations in HIV-1-Infected Drug-Naive Patients from Urban and Suburban Regions of Kenya. *AIDS Res Hum Retroviruses*. 2016;32(3):220–225. MID: 10.1089/aid.2015.0026 PMCID: PMC4779969 26401720 PMC4779969

